# Retinal Diseases Regulated by Hypoxia—Basic and Clinical Perspectives: A Comprehensive Review

**DOI:** 10.3390/jcm10235496

**Published:** 2021-11-24

**Authors:** Ari Shinojima, Deokho Lee, Kazuo Tsubota, Kazuno Negishi, Toshihide Kurihara

**Affiliations:** 1 Laboratory of Photobiology, Keio University School of Medicine, Tokyo 160-8582, Japan; ari.shinojima@keio.jp (A.S.); deokholee@keio.jp (D.L.); 2 Department of Ophthalmology, Keio University School of Medicine, Tokyo 160-8582, Japan; tsubota@tsubota-lab.com (K.T.); kazunonegishi@keio.jp (K.N.); 3 Tsubota Laboratory, Inc., Tokyo 160-0016, Japan

**Keywords:** age-related macular degeneration, angiography, auto-fluorescence, central serous chorioretinopathy, choroidal neovascularization, hypofluorescent, hypoxia-inducible factor, indocyanine green, optical coherence tomography, vascular endothelial growth factor

## Abstract

In recent years, the number of patients with age-related macular degeneration (AMD) is increasing worldwide along with increased life expectancy. Currently, the standard treatment for wet-AMD is intravitreal injection of anti-vascular endothelial growth factor (VEGF) drugs. The upstream of VEGF is hypoxia-inducible factor (HIF), a master regulator of hypoxia-responsive genes responsive to acute and chronic hypoxia. HIF activation induces various pathological pro-angiogenic gene expressions including VEGF under retinal hypoxia, ultimately leading to the development of ocular ischemic neovascular diseases. In this regard, HIF is considered as a promising therapeutic target in ocular ischemic diseases. In clinical ophthalmology, abnormal hypofluorescent areas have been detected in the late-phase of indocyanine green angiography, which are thought to be lipid deposits at the level of Bruch’s membrane to choriocapillaris in vitreoretinal diseases. These deposits may interfere with the oxygen and nutrients that should be supplied to the retinal pigment epithelium, and that HIF/VEGF is highly suspected to be expressed in the hypoxic retinal pigment epithelium, leading to neovascularization. In this review, we comprehensively summarize pathophysiology of AMD-related ocular diseases with the HIF/VEGF pathway from basic and clinic researches with recent findings.

## 1. Introduction

Most living things on earth use oxygen to carry out their life activities. Animals have two major systems that supply oxygen systemically. One is the system of hematopoiesis, which produces the red blood cells which carry oxygen, and the other is the vascular system, which serves as its pathway. The mechanism that protects cells and individuals from the disruption of these systems, i.e., anemia and/or ischemia, is the hypoxic response [1].

When cells are hypoxic, they use the hypoxic response to increase the number of red blood cells that transport oxygen, i.e., hematopoiesis, and the number of blood vessels, i.e., angiogenesis. In addition, they switch energy metabolism to anaerobic metabolism that does not use mitochondria, i.e., metabolic conversion [1].

In 1992, a molecule that is required for cells to increase the hematopoietic factor erythropoietin under hypoxic conditions was discovered and named hypoxia-inducible factor 1 (HIF-1) [2]. It was later discovered that HIF is a master regulator of hypoxia response, controlling not only erythropoietin but also hundreds of genes involved in angiogenesis and metabolic conversion, such as vascular endothelial growth factor (VEGF) [3].

However, abnormalities in the hypoxic response have been found to be a potential factor in a variety of disease formations. In the first part of this review, we introduce the VHL protein (pVHL) encoded by the *VHL* tumor suppressor gene, which is part of the ubiquitin ligase complex that is essential for the specific degradation of HIF, in the context of the actual clinical findings of Von Hippel-Lindau (VHL) disease [4]. The following chapters describe the types and roles of HIFs and the expressions of HIF and VEGF in the eye, especially in the retina.

The number of patients with age-related macular degeneration (AMD) has been increasing worldwide due to the recent increase in life expectancy [5]. The standard treatment for patients with exudative AMD is intraocular injection of anti-VEGF drugs [6,7]. Upstream of VEGF is HIF, and activation of HIF induces expression of various pathological angiogenic genes, including *VEGF*, in the hypoxic retina, ultimately leading to the development of ocular ischemic neovascular diseases [1]. Since HIF is a promising therapeutic target for ocular ischemic diseases, this manuscript includes a chapter on the potential of HIF inhibitors.

In clinical ophthalmology, abnormal hypofluorescent areas are detected in the late phase of indocyanine green angiography in vitreoretinal disease such as AMD [8] or central serous chorioretinopathy (CSC) [9], which are thought to be lipid deposits at the level of Bruch’s membrane to choriocapillaris [9]. These deposits may interfere with the oxygen and nutrients that should be supplied to the retinal pigment epithelium (RPE), and it is strongly suspected that HIF/VEGF is expressed in hypoxic RPE, leading to the formation of neovascularization. Therefore, the actual clinical findings of AMD and CSC will also be presented. The later part of the review also describes pachychoroid neovasculopathy, summarizes the relationship between VEGF and the choroid, and presents actual clinical findings on the background of neovascularization.

Although the underlying causes of choroidal neovascularization (CNV) are complex and multifactorial [10], this review provides a comprehensive description of the pathogenesis of AMD-related ocular diseases involving the HIF/VEGF pathway, including the latest findings from basic and clinical studies. In this review, Pubmed, Japan Medical Abstracts Society, and Google were used to search references.

## 2. Hypoxia-Inducible Factors (HIFs) and Von Hippel-Lindau Disease

VHL disease is a familial cancer syndrome that is dominantly autosomal inherited. The *VHL* gene was elucidated as *VHL* tumor suppressor gene by Latif et al. in 1993 [11]. Hemangioblastomas are the most frequent manifestation of VHL disease. Seventy percent of patients with VHL disease have retinal angiomas, and ocular lesions often precede the development of central nervous system and visceral lesions [12]. Mutations of *VHL* tumor suppressor gene are responsible for the development of a variety of tumors including clear cell renal cell carcinoma (RCC), pheochromocytomas, endolymphatic sac tumors, and pancreatic cysts [13], even though only loss of *VHL* tumor suppressor gene is not enough to induce RCC [14].

**Figure 1:**
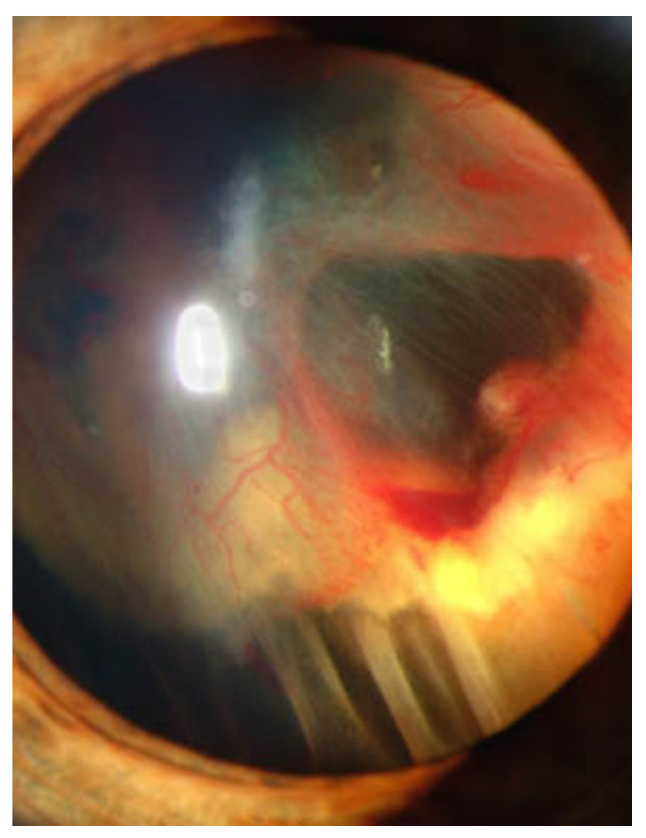
Slit lamp photograph showing retinal detachment in VHL disease. Credit: National Eye Institute, National Institutes of Health Ref#: EDA08 (This image is a work from the National Institutes of Health, a part of the United States Department of Health and Human Services, taken or made as part of an employee’s official duties. As a work of the U.S. federal government, the image is in the public domain).

Under normal oxygen tension, HIF-1α is negatively regulated by proteasomal degradation and ubiquitination in a pathway involving VHL protein (pVHL) encoded by *VHL* gene with one of the recognized components of an E3 ubiquitin protein ligase [4]. As HIF-1α has various roles (metabolisms, angiogenesis, and anti- and pro-apoptosis), dysfunction of *VHL* can induce severe damages in tissues including the eye. The complications of ocular symptoms of dysfunction of *VHL* are subretinal and intravitreal hemorrhage, and progressive fibrovascular proliferation results in tractional detachment [12]. Figure 1 shows the anterior segment of the eye of VHL disease with retinal detachment.

## 3. Types and Roles of HIFs

**Figure 2:**
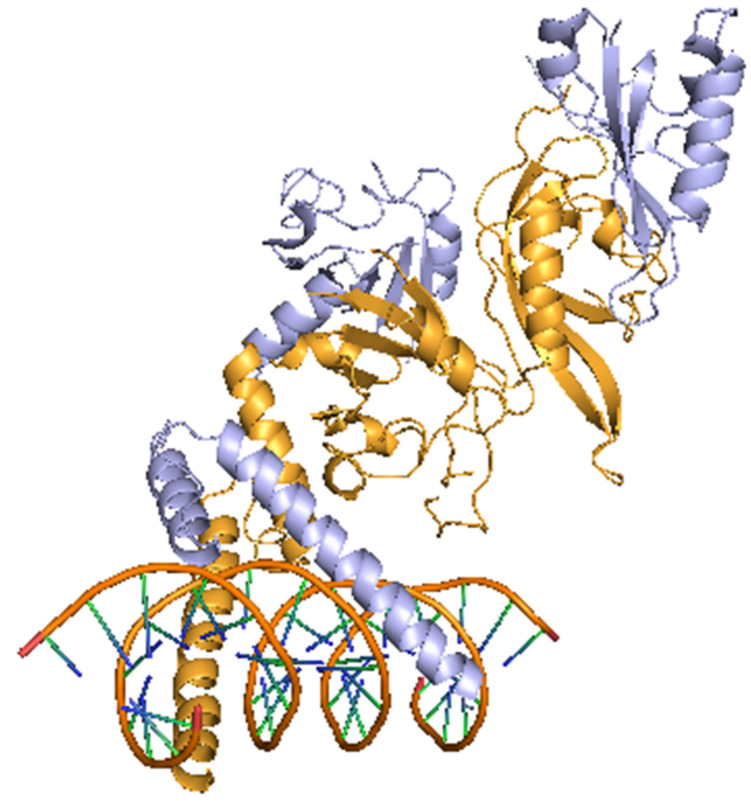
Structure of HIF-1α and HIF-1β. This image is a crystal structure of the heterodimeric HIF-1α and HIF-1β complex. Light yellow color indicates HIF-1α, light purple color indicates HIF-1β, and orange color indicates DNA. Modified from PDB ID: 4ZPR [23].

In 1992, Dr. Semenza and Dr. Wang at Johns Hopkins University discovered a molecule that is necessary for the transcriptional activation of the erythropoietin (*EPO*) gene under hypoxic conditions in a liver cancer cell line (Hep3B), and named it HIF-1 [2,15]. After that, HIFs have become a master regulator of the hypoxic response, controlling not only *EPO* but also hundreds of genes involved in angiogenesis and metabolic conversions, such as VEGF, glucose transporter-1 (GLUT-1), pyruvate dehydrogenase kinase-1 (PDK1), BCL2/adenovirus E1B 19kDa interacting protein-3 (BNIP3), and carbonic anhydrase IX (CA9) [3]. In mammals, in addition to HIF-1α, HIF-2α and HIF-3α are known to exist. These HIFs also have basic helix-loop-helix DNA binding proteins of the PER-ARNT-SIM family (bHLH-PAS) [16]. HIF-2α shares 48 percent sequence identity with HIF-1α [17]. Figure 2 shows structure of HIF-1α and HIF-1β.

*HIF-2α* mRNA is abundantly expressed in organs such as the lungs, heart, and liver under normoxic conditions, while *HIF-1α* mRNA is ubiquitously expressed albeit at a much lower level [18]. Under normoxic conditions, HIF-αs subunits are continuously transcribed and translated. However, when sufficient oxygen concentration is available, the HIF-αs subunits are degraded by the proteasome. A family of prolyl 1-4 hydroxylases (PHD1-4), most prominently PHD2, hydroxylates the oxygen-sensitive α-subunits [19,20,21,22].

**Figure 3:**
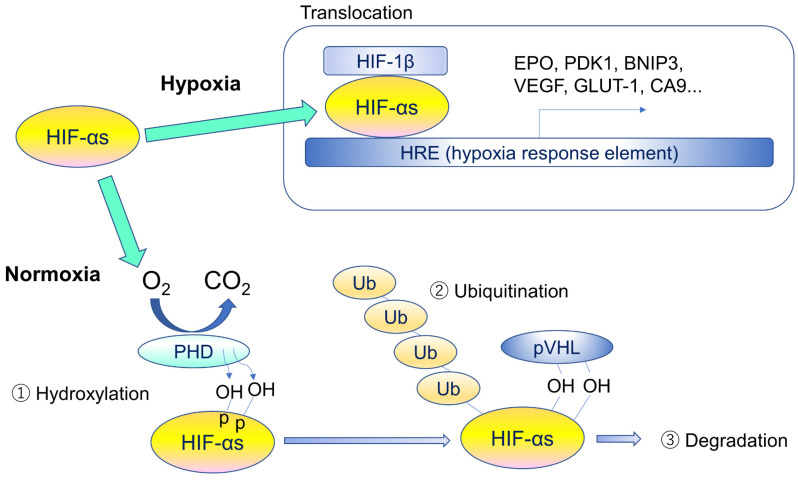
Regulatory mechanism of hypoxia-inducible factors. Under hypoxia, HIF-αs translocate into the nucleus, form heterodimers with β-subunits, and promote transcription of HIF target genes by binding to hypoxia response element (HRE). Under normoxia, specific proline residues of HIF-αs are hydroxylated by prolyl hydroxylase (PHD) (1), and the hydroxylated HIF-αs are ubiquitinated by ubiquitin ligase containing pVHL (2) and degraded by proteasome (3). HIF-αs stands for different forms or multiple α subunits.

Under hypoxic conditions, HIF-αs subunits are no longer polyubiquitylated as functions of PHD and VHL proteins are suppressed by a lack of oxygen. As a result, stabilization of HIF-αs occurs, and stabilized HIF-αs go into nucleus, dimerizes with HIF-1β, and activates hypoxia-responsive gene expressions including VEGF, PDK1, BNIP3, EPO, GLUT-1, and CA9. Lots of genes are involved in pathological angiogenesis, pro- and anti-apoptosis, and metabolic regulations (Figure 3).

In the eye, HIF-1α/SDF-1 pathway and HIF-1α/VEGF pathway have been nominated to induce CNV caused by ocular hypoxia [23]. The relationship between HIF-αs, VEGF and CNV will be discussed in detail in another section.

## 4. Expressions of HIF and VEGF in the Retina

HIFs are not degraded under hypoxia, and *HIFs* regulate angiogenesis with promoting VEGF and *EPO* expressions [24,25]. Flamme et al. reported that the upregulation of *HIF-2α* mRNA in hemangioblastoma, a highly vascularized tumor of the central nervous system, highly correlates with the expression of VEGF in the stromal cells of these tumors [26]. HIFs also regulate the expression of more than 800 genes that are necessary for cells and tissues to survive under hypoxia, including cell proliferation, metabolism, and immunity [27].

The mouse retina is often used to study physiological neovascularization because, unlike the human retina, retinal blood vessels begin to expand after birth. Based on in situ hybridization data on the expression of *Vegf* in the inner layer of the mouse retina, its expression was strongly observed in vascular-free areas and the reduced expression was seen in areas covered by blood vessels [28,29]. We previously generated mice with a retinal neuron specific *Hif-1α* knock-out using the Cre-loxP system, and found that the number of tip cells located at the tip of the stretched blood vessels in the inner retinal layer and the number of filopodia stretched by tip cells were reduced, resulting in a delay in the stretching of retinal blood vessels [30,31]. Furthermore, knock-out of *Hif-1α* specifically in neurons (amacrine cells and horizontal cells) in the mid-retina has been found to result in a sparse vascular bed in the mid-retinal region [32]. On the other hand, astrocyte (a type of glial cell) specific knock-out of *Vegf, Hif-1α* and *Hif-2α*, which line blood vessels in the inner retinal layer, did not change the development of retinal blood vessels [33]. Therefore, the induction of VEGF expression properly in vascular-free areas via HIF, mainly by hypoxic response in neurons, is important in the process of physiological retinal vascular development.

Retinal blood vessels radiate from the optic nerve disc are distributed along the axons of retinal ganglion cells. During the process of building up our body, the blood vessels spread from the proximal end towards the hypoxic area at the distal end. In the premature fundus of low-birth-weight infant, we can observe the process of retinal blood vessel extension in the direction of the vascular-free field at the periphery of the retina. This also shows that the HIFs/VEGF pathway plays an important role in this physiological process of retinal vessel development.

## 5. Retinal Diseases and Anti-VEGF Drugs

As of 2021, in Japan, anti-VEGF drugs are administered intravitreally for AMD with subcentral foveal CNV, macular edema associated with retinal vein occlusion, CNV in pathologic myopia, neovascular glaucoma, and retinopathy of prematurity.

Anti-VEGF drug therapy is the current standard of care in the treatment of neovascular AMD associated with CNV. The injection dose for AMD with subcentral CNV is 0.5 mg (0.05 mL) of ranibizumab (recombinant) or 2 mg (0.05 mL) of aflibercept (recombinant) administered intravitreally every month for 3 consecutive months (induction phase). In the maintenance phase, the interval between doses may be adjusted according to the symptoms, but the interval should be at least one month. Aflibercept is usually administered intravitreally once every two months in the maintenance phase [6,7]. For macular edema associated with retinal vein occlusion or diabetes or CNV in pathologic myopia, ranibizumab at a dose of 0.5 mg (0.05 mL) or aflibercept at a dose of 2 mg (0.05 mL) per dose should be administered intravitreally, with an interval of at least 1 month. In the case of aflibercept injection for diabetic macular edema, 2 mg (0.05 mL) should be administered intravitreally five times consecutively every month, and thereafter, once every two months, with an interval of at least one month depending on symptoms. Ranibizumab, on the other hand, should be administered every month until vision is stabilized. For neovascular glaucoma, a single 2 mg (0.05 mL) dose of aflibercept has been approved for intravitreal administration and, if necessary, re-administration after an interval of at least one month. For retinopathy of prematurity, ranibizumab is administered intravitreally at a dose of 0.2 mg (0.02 mL) once, and may be re-administered, if necessary, but with an interval of at least one month. As shown above, the dosage and the type of anti-VEGF drugs vary slightly depending on the target disease.

## 6. Potential for HIF Inhibitors

We previously reported that an abnormal increase in HIF expression in retinal tissues can promote pathological angiogenesis and exacerbate these diseases in preclinical experiments [34,35].

As previously mentioned in Chapter 4, anti-VEGF drugs are currently the mainstay of treatment for ocular neovascularization. While patients with neovascular AMD undergoing anti-VEGF therapy have been shown to have worsening geographic atrophy [36], this has not been conclusively shown to be an effect of the treatment itself and may be due to underlying disease progression. 

Fallah et al. summarized phase II/III clinical trials of HIF inhibitors in cancer treatment [37]. However, there have been no clinical trials of HIF inhibitors for retinal diseases. HIF inhibitors in natural foods themselves are being studied as follows.

Ibuki et al., discovered several HIF inhibitors through extensive screenings of food ingredients, and found that a diet containing these ingredients was effective in reducing the risk of laser-induced CNV in mice [38,39,40]. Furthermore, treatment of these ingredients/compounds reduced *Vegf* mRNA expression as well as HIF activation under CoCl_2_-induced pseudo hypoxic conditions [38,39,40]. Otherwise, Lee et al. found that resveratrol significantly reduced the expression of *HIF-1α* and *VEGF-A* in human ARPE19 cells and CNV mouse models by inhibiting the PI3K/AKT/mTOR signaling pathway, which promotes proteasome-mediated degradation of HIF-1α. As a result, CNV volume was found to be reduced [41]. Shoda et al. found that some of 82 water-soluble extracts from marine products inhibited HIF expression and significantly suppressed pathological angiogenesis in the retina by about 65% in a mouse model of oxygen-induced retinopathy, which mimics diabetic retinopathy and retinopathy of prematurity [42]. In addition, angiogenic factors such as *Vegf* and *Epo*, which are target genes of HIFs, were also significantly suppressed by the administration of water-soluble fish extracts [42]. 

Foods or food extracts that inhibit HIF, which shows a significant decrease in the amount of CNV in mice when administered orally are shown in Table 1.

As a non-pharmacological approach, Koo et al., used therapeutic gene editing in a mouse model of CNV to intravitreally inject the CRISPR RNA-induced endonuclease LbCpf1 (from Lachnospiraceae bacterium ND2006) targeting *Hif-1α* or *Vegfa*. This reduced the volume of CNV with the same efficiency as aflibercept [43].

Not only marine products, but also mushroom products can significantly inhibit HIF activation. Lee et al., demonstrated that 2-azahypoxanthine (AHX), a fairy chemical from the fungus Lepista sordida [44,45], had an inhibitory effect on HIF activation in retinal cells and suppressed *Vegf* mRNA upregulation under CoCl_2_-induced pseudo hypoxic conditions [46]. Moreover, Kunimi et al., reported inhibition of the *Hif-1α/Bnip3* pathway has a retinal neuroprotective effect [47,48,49]. Although the clinical study using HIF-inhibitors has not been started, a HIF inhibitor becomes available in the future, it would have the advantage of inhibiting VEGF in addition to retinal protection. Future studies will need to test these hypotheses.

## 7. Age-Related Macular Degeneration (AMD)

The number of patients with AMD has been on the rise worldwide in recent years as life expectancy increases [5]. The standard treatment for patients with exudative AMD is intraocular injection of anti-VEGF drugs, while HIF is upstream of VEGF. In this section, the relationship between VEGF and HIF will be discussed from actual cases.

The characteristics of dry AMD is the atrophy typically involving the choriocapillaris, RPE, and photoreceptor elements (rods and cones), and it does not involve leakage of the blood or serum. On the other hand, wet AMD characterized by exudative changes including serous or hemorrhagic detachment of RPE and CNV, which lead to leakage and fibrovascular scarring [50]. Rim et al., reported a prospective link between cigarette smoking and subsequent risk of neovascular AMD among Asian men and concluded that a dose-dependent relationship exists between the duration and intensity of smoking and risk of neovascular AMD [51]. 

**Figure 4:**
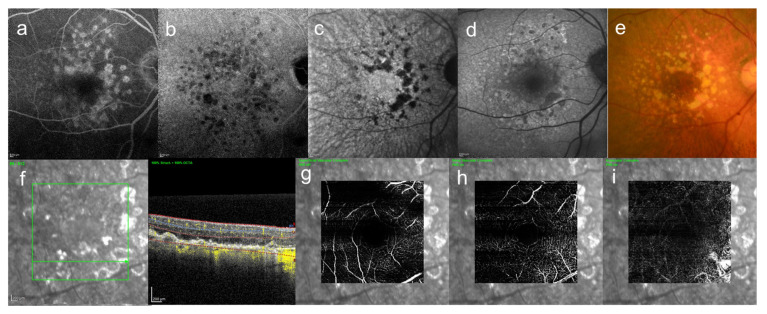
An 85-year-old woman with AMD. These images are the right eye with 20/16. This patient came to the hospital with a complaint of contralateral gradual loss of vision 4 months ago. No other systemic diseases. (**a**) FA image acquired at 16 min, increasing fluorescence throughout the angiogram but the margins remain distinct, (**b**) ICGA image, abnormal hypofluorescence contrast became clearer acquired at 26 min, (**c**) near-infrared autofluorescence image, abnormal hypofluorescence can be seen broadly which corresponds to the hypofluorescent area of ICGA images, (**d**) short-wavelength fundus autofluorescence image, abnormal hyperfluorescence and confluent hypofluorescence can be seen broadly, (**e**) color fundus photograph, drusen and geographic atrophy can be seen broadly, (**f**) infrared + OCT angiography (horizontal section) through the drusen, (**g**) Superficial vascular segment OCT angiography, (**h**) Deep vascular segment OCT angiography, (**i**) Avascular segment OCT angiography, (**g**–**i**) where there is no macular neovascularization, but enlarged choroidal vessels (**i**).

**Figure 5:**
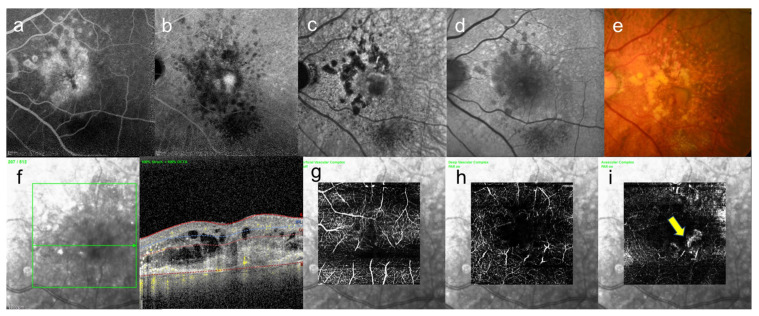
Same case as Figure 4. These images are left eye with 20/250. This eye is contralateral eye of above figures. (**a**) leakage became enlarged gradually on FA image acquired at 15 min, and (**b**) abnormal hypofluorescence and leakage became enlarged on ICGA image acquired at 25 min, and leakage can be seen around the fovea, (**c**) near-infrared autofluorescence image, abnormal hypofluorescence can be seen broadly which corresponds to the hypofluorescent area of ICGA images, (**d**) short-wavelength fundus autofluorescence image, abnormal hyperfluorescence and hypofluorescence can be seen broadly, (**e**) color fundus photograph, drusen and geographic atrophy can be seen broadly, (**f**) infrared + OCT angiography (horizontal section) through the fovea, (**g**) superficial vascular segment OCT angiography, (**h**) deep vascular segment OCT angiography, (**i**) avascular segment OCT angiography, where there is macular neovascularization (arrow).

Large drusen (>63 μm) are often a precursor of late AMD [52], but eyes without large drusen can also develop CNV [53]. In our actual cases, drusen was seen in both eyes, but CNV appeared only in one eye, causing hemorrhage and exudation, and retinal edema (Figure 4 and Figure 5).

As shown in Figure 4 and Figure 5, drusen are scattered throughout the macula, but there is a significant difference in visual acuity depending on whether neovascularization is present and where it is located.

In 2010, the results of two independent genome-wide association studies (GWASs) identified several new genes associated with advanced AMD status [52,54]. Chen et al. identified a susceptibility locus near TIMP3 (tissue inhibitor of metalloproteinase 3), which is a metalloproteinase involved in degradation of the extracellular matrix and previously implicated in early-onset maculopathy [54]. These studies implicated genes associated with lipid metabolism, specifically the HDL pathway, ABCA1, LIPC, CETP, and LPL [54,55]. Lorés-Motta et al. showed localization of complement-factor-H-related (CFHR) genes in the choriocapillaris and in drusen. Thus, CFHR proteins are key proteins in the AMD disease mechanism. Therapies that modulate CFHR proteins might be effective for treating or preventing progression of AMD. Therefore, not only HIF-targeting therapies, such therapies could target specific individuals with AMD based on their genotypes at the CFH locus [56]. Anti-VEGF therapy is known to effectively improve and maintain vision in most cases of advanced wet AMD [57,58,59], however repeated anti-VEGF injections may increase the risk of ocular and systemic complications [60,61]. Those with extensive intermediate size drusen, at least 1 large drusen, noncentral geographic atrophy in 1 or both eyes, or advanced AMD (photocoagulation or other treatment for CNV, or GA involving the center of the macula, non-drusenoid RPE detachment, serous or hemorrhagic retinal detachment, hemorrhage under the retina or the RPE, and/or subretinal fibrosis) or vision loss due to AMD in 1 eye, and without contraindications such as smoking, should consider taking a supplement of antioxidants plus such as that used in AREDS study [62]. Clinically, it was known that abnormal hypofluorescent lesions are identified in late ICGA findings of AMD [8]. However, AMD can be associated with complex findings such as hemorrhage and neovascularization that block fluorescence. In 2016, Shinojima et al. used Enface OCT to determine where in the fundus the abnormal hypofluorescent areas identified in late ICGA of CSC were localized [9], and found that they were at the level of the Bruch’s membrane to choriocapillaris, and thought to be related to lipid deposition. Lipid deposition from Bruch’s membrane to choriocapillaris suggests that the lesions are hypoxic. The products of lipid peroxidation can accumulate lipofuscin which is a heterogenous protein-lipid-carbohydrate aggregate [63], and reduce activity of RPE autophagy [64]. 

When the supply of oxygen and nutrients from the choroidal side to the retinal side is inhibited, hypoxia-inducible factors such as HIF-1α appear and generate cytokines such as VEGF, which could cause CNV. Otherwise, it has been speculated that a complex combination of pathological excessiveness of reactive oxygen species and oxidative stress and dysfunctional (insufficient) autophagy pathways in the aged AMD RPE cells results in CNV [10].

## 8. Central Serous Chorioretinopathy

CSC is characterized by the presence of a serous retinal detachment associated with leakage, RPE alterations and increased choroidal thickness [65,66] The onset of CSC is generally in the 30s–50s and sometimes unilateral, sometimes bilateral [67,68]. Acute CSC is unilateral in most cases. Indeed, at initial presentation, bilateral CSC has been reported in only 5% to 18% of cases [69,70].

Imamura et al. reported that the choroidal thickness in CSC is significantly greater than that in normal eyes using enhanced depth imaging (EDI) of spectral-domain optical coherence tomography (OCT) [71]. Even in many sample sizes, subfoveal choroidal thickness is significantly greater than that in healthy eyes in both acute and chronic CSC [72]. Scleral thickening [73] and asymmetric vortex veins [74] have been reported in recent years as causes of choroidal thickening. Spaide et al. suggested the venous outflow from the choroid may be modulated according to the passage of the vortex veins through the sclera to form a Starling resistor [75].

**Figure 6:**
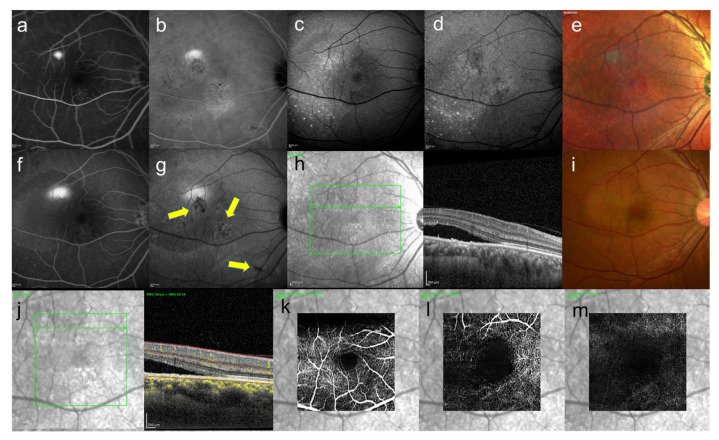
39-year-old man with unilateral CSC. These images are images of CSC. This patient came to the hospital because of difficulty in vision of his right eye from a month ago. The symptoms disappeared spontaneously four months after the first visit, but recurred five months later. (**a**) FA image acquired at 4 min, smokestack pattern leakage can be seen and (**b**) ICGA image acquired at 12 min, leakage and hypofluorescent area can be seen (**c**) short-wavelength fundus autofluorescence image, (**d**) near-infrared autofluorescence image, partial hyper- and hypofluorescent area can be seen, (**c**,**d**) discrete small dots with hyperautofluorescence can be seen, (**e**) multi-color fundus photograph, the color tone of the leaking area is different from others, (**f**) FA image acquired at 18 min, smokestack pattern leakage enlarged and (**g**) ICGA image acquired at 26 min, abnormal hypofluorescence can be seen (arrows) (**h**) infrared + OCT (horizontal section) through the serous retinal detachment, (**i**) color fundus photograph, serous retinal detachment with fibrinous contents were observed, (**j**) infrared + OCT angiography (horizontal section) through the serous retinal detachment, (**k**) superficial vascular segment OCT angiography, (**l**) deep vascular segment OCT angiography, (**m**) avascular segment OCT angiography, (**k**,**l**) there is no macular neovascularization.

In clinical practice, it is necessary to speculate the pathology of fundus diseases based on images obtained by autofluorescence, fluorescence angiography (FA) and OCT to consider treatment. As mentioned earlier, in 2016, Shinojima et al. used Enface OCT to determine where the abnormal hypofluorescent areas identified in late-phase ICGA of CSC were localized in the fundus [9]. We found that the areas showing abnormal hypofluorescence in late-phase ICGA correlated with abnormal hyperreflective areas at the level of the choriocapillaris to Bruch’s membrane under the RPE in Enface OCT, and we thought that they were related to lipid deposition. We also confirmed that all CSC and some of their contralateral eyes showed abnormal hypofluorescent areas in late-phase ICGA, and some of the contralateral eyes showed serous retinal detachment during follow-up [76]. Thus, the abnormal hypofluorescent areas seen in late-phase ICGA cannot be ignored in considering the pathogenesis of CSC. We present a representative case in Figure 6. 

It is important to note that hypofluorescent area can be seen in late ICGA without necessarily having drusen like AMD, as shown in the color photo in Figure 6.

We hypothesize that lipid deposition at the level of the choriocapillaris to the Bruch’s membrane under the RPE inhibits the supply of oxygen and nutrients from the choroidal side to the retinal side, causing the appearance of hypoxia-inducible factors such as HIF-1α and the generation of cytokines such as VEGF, leading to abnormal morphology of the choriocapillaris [77], which in turn leads to thickening of the choroid in CSC.

The choroid is around 500 µm in some normal healthy eyes [78]. Even if the choroid is thickened, leakage will not occur if the RPE barrier has not been disrupted. Photocoagulation therapy works by sealing the disrupted RPE [79]. About 90% of CSC eyes had complete resolution of the SRD at 12 months after the half-dose verteporfin photodynamic therapy [80]. We reported that when we observed the local abnormal hypofluorescent area over time in the late-phase ICGA, the area expanded with time [81], and that the area decreased after photodynamic therapy [82]. Based on these reports, we believe that improvement of this presumed lipid deposition will improve the pathology. However, CSC is multifactorial. We have recently reported that near-infrared autofluorescence correlates well with the hypofluorescent area of late-phase ICGA [83], and we believe that multimodal imaging will allow us to speculate the pathogenesis in more detail and lead to better treatment.

## 9. Pachychoroid Neovasculopathy

The term “pachychoroid” means “thickened choroid” [84]. CSC is one of the spectrums of pachychoroid disease. The presence of hypertrophic or congested vessels in the choroid (pachyvessels), under an area of reduced or absent choriocapillaris in the posterior pole, appears to be the typical feature of pachychoroid. As other features, there are ICGA hyperfluorescence, blood-flow signal attenuation within the choriocapillaris and inner choroid by OCT angiography, RPE layer alterations and thinning of the outer nuclear layer, and the presence of pachydrusen [84]. The clinical spectrum of pachychoroid disease may be subdivided as follows: “disorders with exudative changes”, “disorders with neovascularization” and “disorders with atrophic changes” [84].

**Figure 7:**
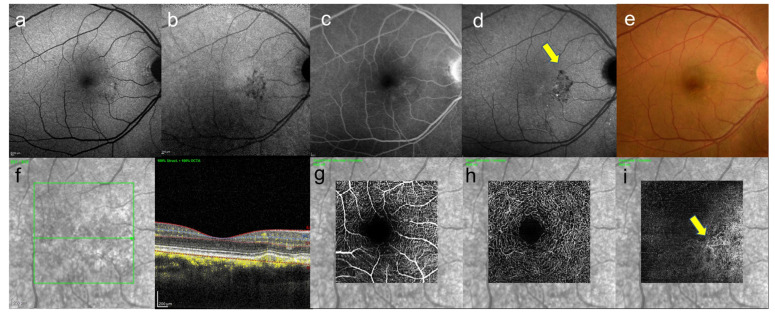
A 46-year-old man with unilateral CSC. These images are the eye without serous retinal detachment, contralateral eye. (**a**) short-wavelength fundus autofluorescence image, hyperautofluorescence and hypoautofluorescence near the fovea can be seen, (**b**) near-infrared autofluorescence image, hypoautofluorescence near the fovea can be seen, (**c**) FA image acquired at 22 min, slight staining was observed and (**d**) ICGA image acquired at 27 min, abnormal hypofluorescence can be seen (arrows) which corresponds to the hypofluorescent foci in near-infrared autofluorescence image, (**e**) color fundus photograph, pigmentation irregularity can be seen, (**f**) infrared + OCT (horizontal section) through the fovea. There is no serous retinal detachment but flat irregular RPE is found, (**g**) superficial vascular segment OCT angiography, (**h**) deep vascular segment OCT angiography, (**g**,**h**) there is no macular neovascularization, (**i**) avascular segment OCT angiography, where there is macular neovascularization (arrow).

Here, we introduce one case of pachychoroid neovasculopathy which is one of the spectrums pachychoroid disease (Figure 7). In this case, one eye had been followed up as CSC for almost 20 years and the contralateral eye had never appeared serous retinal detachment and had been followed up, hypofluorescent foci in late-phase ICGA (Figure 7b), where there were slight abnormal blood vessels in the area of hypofluorescence of ICGA were confirmed by OCT angiography (Figure 7i).

This is a case in which abnormal blood vessels were not detected by FA or ICGGA, but were detected by OCTA. Although cases of CSC with development of CNV have been reported in the past [85,86], such cases are likely to increase in the future as life expectancy increases.

## 10. VEGF and the Choroid

VEGF is physiologically secreted from the RPE to maintain choroidal homeostasis [87,88]. Shinojima et al., reported that lipid deposition at the level of the Bruch’s membrane to choriocapillaris can be detected clinically from en-face OCT and ICGA imaging [9,77]. It has been suggested that the thickening of Bruch’s membrane may interfere with this RPE and choriocapillaris paracrine relationship and prevent the passage of VEGF-A partially from the RPE to the choriocapillaris during hypoxia and aging [87,89,90]. This could result in a VEGF imbalance, which could trigger the disease. In autopsies of normal eyes, it was found that the diameter of the choriocapillaris and the thickness of the choroid decreased from 10 to 100 years of age, while the thickness of Bruch’s membrane increased with age [91].

**Figure 8:**
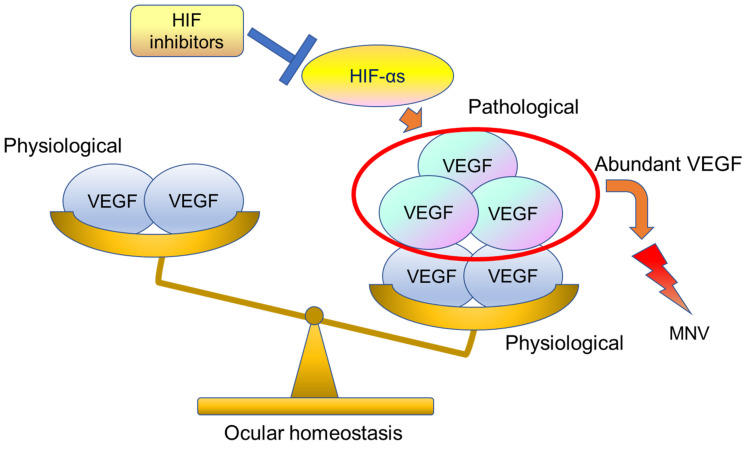
This image shows ocular homeostasis. Usually, physiologically VEGF-A is secreted by the RPE and exerts a trophic influence on the choriocapillaris. However, abundant HIF-αs can cause induction of pathological levels of VEGF and lead to macular neovascularization (MNV). HIF inhibitors can suppress abundant VEGF. HIF-αs stands for different forms or multiple α subunits.

In RPE-specific *Vhl* knock-out mice, atrophy of photoreceptor and RPE cells and abnormal dilatation of choriocapillaris have been observed [92]. On the other hand, astrocyte- and RPE-specific knock-out of *Hif-2α* has been found to inhibit pathological angiogenesis in the retina and choroid, respectively, to the same extent as *Vegf* knock-out [33,34]. Surprisingly, RPE-specific *Vegf* knock-out mice show atrophy and loss of function in choriocapillaris and cone cells within a few days after gene deletion, whereas both *Hif-1α* and *Hif-2α* knock-out mice show no physiological abnormalities [34]. This means that *Vegf* expression in the steady state is necessary not only for the maintenance of existing blood vessels but also for neuronal activity. If VEGF is suppressed too much, ocular homeostasis balance is collapsed. In fact, during large-scale clinical trials, it has been reported that atrophy of the RPE and photoreceptor cells can be observed after long-term treatment with anti-VEGF therapy [36]. On the other hand, abundant VEGF can cause ocular homeostasis imbalance, which can cause macular neovascularization. Therefore, physiological balance is needed for our healthy ocular homeostasis (Figure 8).

HIF-α is a protein that is degraded under normal oxygen and does not exist after the developmental period, which means that it is not required for retinal homeostasis in the steady state [1]. We assume that HIF may be a more ideal therapeutic target to control pathophysiological hypoxic responses.

## 11. Conclusions

In this review, we described that HIFs may induce pathological pro-angiogenic gene expressions including *VEGF* under retinal hypoxia, ultimately leading to the development of ocular ischemic neovascular diseases. If HIFs inhibitors become available in the future, they may have the advantage of inhibiting VEGF in addition to protecting the retina. We also suggested that the HIF/VEGF pathway may need to be considered when looking at pachychoroid spectrum diseases associated with neovascularization. We hope that this review will serve as a bridge between clinical and basic research.

## Figures and Tables

**Table 1:** The list of foods or extracts that inhibit HIF showing a significant decrease in the amount of CNV in mice.

Representative HIF Inhibitors	References
Garcinia cambogia extract	Ibuki, et al. [38]
Hydroxycitric acid	Ibuki, et al. [38]
Lactoferrin	Ibuki, et al. [39]
Rice bran	Ibuki, et al. [40]
Vitamin B6 (pyridoxine hydrochloride)	Ibuki, et al. [40]
Resveratrol	Lee, et al. [41]
Selar crumenophthalmus	Shoda, et al. [42]
Seriola dumerili	Shoda, et al. [42]
Spratelloides gracilis	Shoda, et al. [42]
Decapterus macarellus	Shoda, et al. [42]
Decapterus tabl	Shoda, et al. [42]
Decapterus muroadsi	Shoda, et al. [42]

## Data Availability

The data presented in this study are available on request from the corresponding author. The data are not publicly available due to patients’ privacy.
